# Polyclonal serum free light chain elevation is associated with increased risk of monoclonal gammopathies

**DOI:** 10.1038/s41408-019-0210-z

**Published:** 2019-05-17

**Authors:** Shaji Kumar, Dirk R. Larson, Angela Dispenzieri, Terry M. Therneau, David L. Murray, P. Leif Bergsagel, Robert A. Kyle, S. Vincent Rajkumar

**Affiliations:** 1 Division of Hematology, Mayo Clinic, Rochester, MN USA; 2 Department of Health Sciences Research, Mayo Clinic, Rochester, MN USA; 3 Department of Laboratory Medicine and Pathology, Mayo Clinic, Scottsdale, AZ USA; 4 Department of Hematology/Oncology, Mayo Clinic, Scottsdale, AZ USA

**Keywords:** Epidemiology, Oncogenesis, Risk factors

## Abstract

Monoclonal gammopathies (MG) constitute a spectrum of disorders starting from a monoclonal gammopathy of undetermined significance (MGUS) to active disease requiring therapy such as multiple myeloma. MG are characterized by proliferation of clonal plasma cells (PC) secreting a monoclonal protein either as intact immunoglobulin or free kappa or lambda free light chains (FLC). We hypothesized that a polyclonal elevation of serum FLC may indicate an inflammatory state that precedes development of MG. We studied 15,630 individuals from Olmsted county, who did not have MGUS based on baseline screening studies. At a median follow-up of 18.1 years, 264 patients had developed a clonal PC disorder; 252 with MGUS, 1 with SMM, 8 with MM, and 3 with amyloidosis, translating to an annual incidence of development of a MG of 0.1%. We examined the baseline polyclonal ΣFLC (kappa + lambda FLC) from the initial screening and grouped them into deciles. The highest decile group had a 2.6-fold (95% CI; 1.8, 3.7) increase in the risk of developing a MG, *P* < 0.001. We demonstrate for the first time, the increased risk of developing MG in patients with elevated serum FLC, suggesting that an underlying inflammatory state may play an etiologic role.

## Introduction

Monoclonal gammopathy of undetermined significance (MGUS) is a relatively common abnormality in the older individuals, characterized by clonal proliferation of mature plasma cells in the bone marrow, with small numbers of these cells observed in the peripheral blood.^1^ The prevalence is estimated to be 3.2 per 100,000 individuals greater than 50 years of age; increasing in prevalence with age with 5.3 per 100,000 among those over 70 years of age.^2^ While the condition itself is asymptomatic and with no clear consequence, the significance lies in the increased risk of development of a plasma cell disorder requiring therapy such as multiple myeloma or amyloidosis or another related lymphoid disorder.^3–5^ Large epidemiological studies of MGUS from various parts of the globe have estimated this risk to be ~1% per year, with no change in risk over the years. Various risk factors have been identified for identifying patients who are at a higher risk for progression, including the level and type of the monoclonal protein, and abnormal serum free light chain ratio.^6^

While much has been studied regarding the risk of progression from MGUS, there is limited information regarding the risk factors for development of MGUS.^7^ There are clearly genetic and environmental factors that predisposes one to the development of monoclonal gammopathies. Increased risk of MGUS have been found among the first degree relatives of those with a diagnosis of myeloma, and genetic loci have been identified that are associated with an increased risk of development of myeloma.^8^ Environmental factors such as exposure to ionizing radiation, petroleum products, and pesticides have been implicated in the development of myeloma and likely influences the development of MGUS as well.^9^ Chronic infection has been proposed as a risk factor for development of MGUS, but the reduced prevalence seen in the Asian population may argue against this possibility. It is important for us to identify factors that can predict the development of monoclonal gammopathies, as this can potentially give us the opportunity to explore the prevention approaches.

Plasma cells, normal as well as clonal, secrete small amounts of kappa or lambda free light chains and these are quickly eliminated by the kidneys resulting in relatively low levels of circulating serum free light chains. However, in the presence of clonal plasma cells as with MGUS or MM, there is excess production of one or other light chain leading to a skewed ratio between the two light chains, as well as overall increased levels.^10^ An abnormal serum free light ratio becomes more common along the spectrum of disease progression from MGUS to SMM and finally to active MM.^11^ A polyclonal elevation of both kappa and lambda serum free light chain can be observed in many individuals, not resulting in an abnormal FLC ratio, the etiology of which is not clear. It has been observed in the context of immune activation as it can occur with chronic infections and inflammatory states.

We hypothesized that a polyclonal increase in serum free light chain may help to identify individuals at higher risk of developing a monoclonal gammopathy. We undertook this study in a population-based cohort who have been previously examined for the prevalence of MGUS to ascertain if increased polyclonal FLC levels were associated with an increase in the risk of MGUS and related disorders including smoldering multiple myeloma (SMM), multiple myeloma (MM), or light chain amyloidosis.

## Patients and methods

### Patient selection

We used a population-based cohort previously assembled by us to estimate the prevalence of MGUS.^1,2,5^ The original cohort comprised 21,463 out of the 28,038 enumerated Olmsted County residents aged 50 years or older, as of January 1, 1995, in whom blood samples were available for study. From among the 21,462 subjects in the Olmsted County MGUS prevalence cohort, we excluded the 4096 anonymized subjects, 710 subjects who were known to have a plasma cell disorder (MGUS or light chain MGUS), 758 subjects whose remaining sample was insufficient to perform the FLC assay, 52 subjects who were not Mayo Clinic patients, and 216 subjects who did not have a plasma cell disorder but had an abnormal FLC ratio. This resulted in a cohort of 15,630 patients who had no evidence at baseline of a clonal plasma cell disorder. We obtained the FLC results on this cohort from samples collected at baseline when the Olmsted prevalence study was conducted.^10^

### Laboratory methodology

Following approval by the Mayo Clinic Institutional Review Board, the FLC assay (FREELITE™, The Binding Site Ltd., Birmingham, UK) was measured in the 18,372 stored serum samples on a Dade Behring BNII automated nephelometer (Siemens, Newark, Delaware). In addition to reporting the κ and λ FLC concentrations, the assay reports the FLC κ/λ ratio (FLC-R), and an abnormal result was defined as an abnormal FLC-R (normal diagnostic range: 0.26–1.65). As described earlier, we excluded all patients with MGUS and all patients with an abnormal serum FLC ratio at baseline from the study cohort. We used the sum of the kappa and lambda FLC (ΣFLC) to examine the impact of polyclonal elevation of serum free light chains. We compared the risk of development of a clonal plasma cell disorder during follow-up among patients in the top decile (10th decile) of ΣFLC compared with the lower 9 deciles (1–9th deciles).

### Statistical analysis

For the primary analysis, we identified all patients from the study cohort who developed a clonal plasma cell disorder including SMM, MM, and immunoglobulin light chain amyloidosis during the course of follow-up through electronic search of the Mayo Clinic Dysproteinemia database. We abstracted all relevant clinical and laboratory data needed for the study from the electronic medical records. Rates of developing a monoclonal gammopathy were computed using the Kaplan–Meier method; patients were followed until the diagnosis of a monoclonal gammopathy (MGUS, smoldering myeloma, multiple myeloma, or primary amyloid), or until the date of the last negative serum protein electrophoresis (SPE). Progression rates were depicted graphically using Kaplan–Meier curves. The risk of developing a monoclonal gammopathy associated with an increased polyclonal FLC was evaluated using Cox proportional hazards regression models. Hazard ratios for patients with a ΣFLC in the highest decile relative to those in the lower 9 deciles were reported with 95% confidence intervals. Follow-up was summarized using the median time to censoring; this is based on a Kaplan–Meier estimate in which the deaths were censored, and those alive at last follow-up were counted as events. All analysis was performed using SAS version 9.4 (SAS Institute Inc., Cary, NC) and R version 3.3.1 (R Core Team (2016), Foundation for Statistical Computing, Vienna, Austria).

## Results

The final cohort consisted of 15,630 individuals from Olmsted county who did not have MGUS or light chain MGUS following baseline screening studies; 8631 (55.2%) were females and the median age was 63 years (range: 50, 109). The median follow-up of the cohort was 18.1 years (range from 0 to 19. 9 years). The laboratory characteristics at the time of initial work-up is shown in Table 1.

**Table 1:** Laboratory characteristics at initial work up

	All patients (*n* = 15630)	ΣFLC Decile 10 (*n* = 1560)	ΣFLC Decile 1–9 (*n* = 14,070)
Age	63 (50, 109)	73 (50, 109)	62 (50, 101)
Age >70 (*n*, %)	4366 (28%)	896 (57%)	3470 (25%)
Gender: male (*n*, %)	6999 (45%)	769 (49%)	6230 (44%)
Serum free kappa (mg/dL)	1.3 (0.05, 20.5)	2.8 (1.1, 20.5)	1.2 (0.1, 101.0)
Serum free lambda (mg/dL)	1.5 (0.04, 28.2)	3.1 (1.8, 28.2)	1.5 (0.04, 3.6)
Kappa/lambda ratio	0.8 (0.3, 1.6)	0.9 (0.3, 1.6)	0.8 (0.3, 1.6)
ΣFLC (mg/dL)	2.8 (0.09, 48.2)	5.8 (4.7, 48.2)	2.7 (0.09, 4.73)
Serum creatinine^a^ (mg/dL)	1.0 (0.4, 14.2)	1.2 (0.4, 14.2)	1.0 (0.4, 10.8)

*Note*: All values are median, (min, max) unless otherwise noted

^a^Based on 12,944 patients with serum creatinine

During the follow up period, 264 patients developed a clonal plasma cell disorder; 252 with MGUS, 1 with SMM, 8 with MM, and 3 with amyloidosis. This translated to an annual incidence of development of one of the disorders of 0.1% (264 events over 221,648.65 person-years of follow-up).

**Fig. 1:**
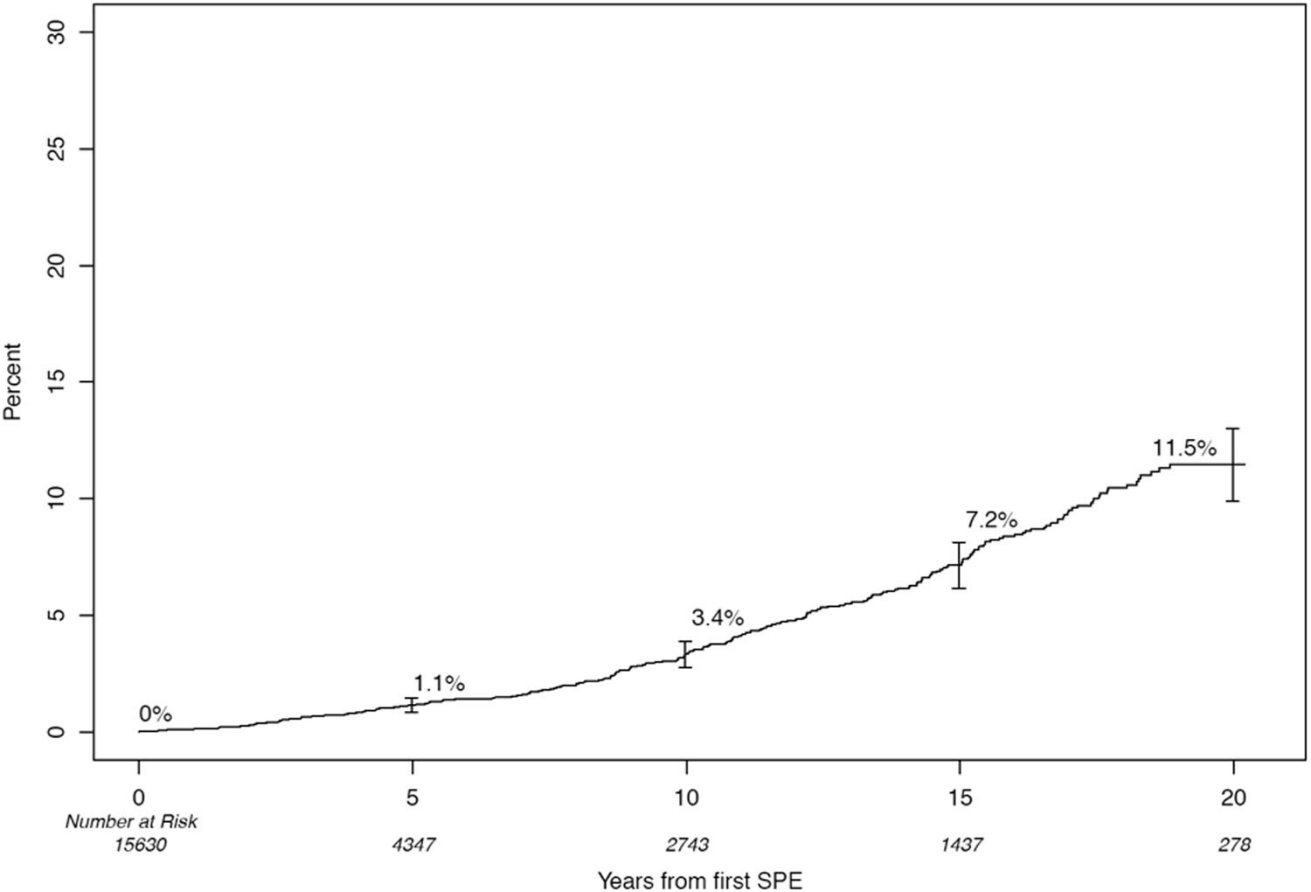
Cumulative risk of development of any monoclonal gammopathy (MGUS, SMM, or MM) among those in the highest decile of FLC

**Fig. 2:**
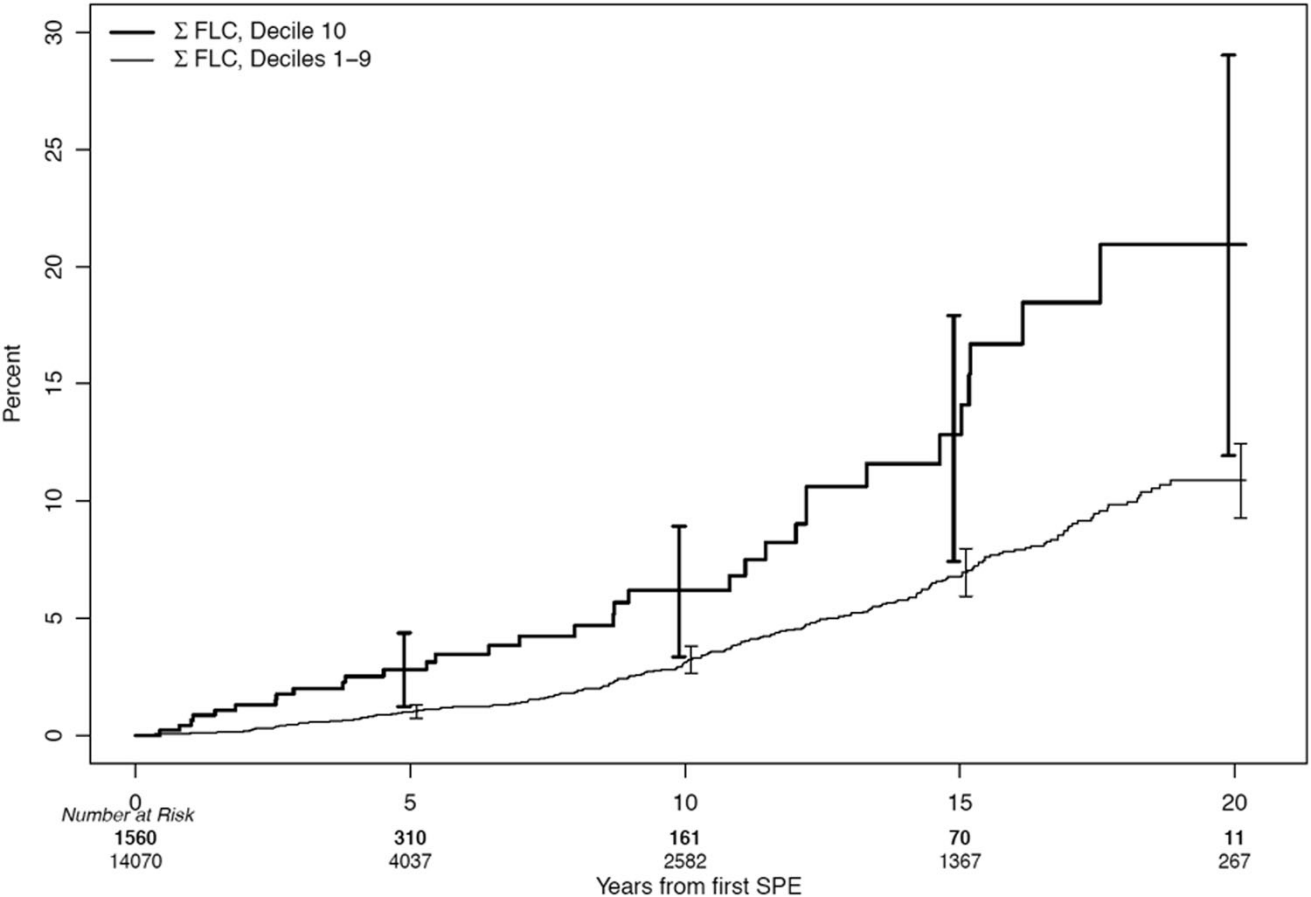
Cumulative risk of development of MGUS, SMM, or MM among those in the highest decile of FLC

We first examined the baseline polyclonal ΣFLC obtained at the time of initial screening for the study cohort and grouped them into deciles. We identified 1560 patients with ΣFLC level in the highest decile (Table 2). We then examined if increased polyclonal ΣFLC levels in the highest decile were associated with an increase in the risk of MGUS and related disorders including SMM, MM, and immunoglobulin light chain amyloidosis compared with patients in the lower 9 deciles. Using the last date of follow-up for censoring, the highest decile group had a 2.6-fold (95% CI; 1.8, 3.7) increase in the risk of developing a monoclonal gammopathy, *P* < 0.001. Using the last known date of negative SPE for censoring, the risk of developing monoclonal gammopathy was 2.2 (1.5, 3.2), *P* < 0.001. The cumulative risk of development of any monoclonal gammopathy over time is shown in Fig. 1, and the risk of development of MGUS, SMM, or MM is shown in Fig. 2.

**Table 2:** Risk of developing a monoclonal gammopathy

Censoring	Group	*N*	Events	2-year rate (95% CI)	5-year rate (95% CI)	10-year rate (95% CI)	15-year rate (95% CI)	HR (95% CI)	*P*-value
Last negative SPE^a^	Overall	15,630	264	0.3% (0.1, 0.4)	1.2% (0.9, 1.4)	3.3% (2.8, 3.9)	7.2% (6.2, 8.1)	NA	
	ΣFLC Decile 1–9^b^	14,070	231	0.2% (0.1, 0.3)	1.0% (0.7, 1.3)	3.1% (2.5, 3.7)	6.8% (5.8, 7.8)	1.0	
	ΣFLC Decile 10^c^	1560	33	1.3% (0.3, 2.3)	2.8% (1.2, 4.4)	6.2% (3.3, 8.9)	12.8% (7.4,17.9)	2.3 (1.6, 3.3)	<0.001

^a^Subjects who did not develop a plasma cell disorder were censored at the date of their last negative SPE. Of the 15,630 subjects, 5273 had a subsequent SPE after the screening study

^b^ΣFLC ≤ 4.73

^c^ΣFLC > 4.73

## Discussion

Much has been learned in terms of the risk factors for progression from MGUS to smoldering multiple myeloma and active multiple myeloma. It is well-known that all patients with myeloma have a preceding phase of MGUS that can exist upto 35 years before patients develop active disease.^1,12,13^ Less is known regarding the development of MGUS, in terms of risk factors and most importantly biomarkers for its development in normal individuals. Epidemiological studies have suggested multiple risk factors for development of monoclonal gammopathies, including exposure to hydrocarbons, pesticides, and radiation.^9,14–18^ A genetic predisposition has been highlighted by the increased prevalence of MGUS in the African–American population, who has a 2 to 3 full higher risk compared with Caucasians.^19–21^ In addition, familial studies have demonstrated a higher risk of MGUS in first-degree relatives of patients with myeloma and other monoclonal gammopathies.

There is very little information on the specific inciting factors that lead to the development of the clonal plasma cell population. Chronic antigenic stimulation has been incriminated for a long time in the development of monoclonal gammopathies in general and myeloma in particular, with a variety of different causative factors being suspected, including infectious organisms such as herpes virus, as well as a variety of other chronic infections.^22,23^ Detailed analysis of the involved isotype in patients with myeloma has suggested presence of antibodies against a variety of proteins including autoantigens and infectious organisms. In one study, paraproteins from 29 of 192 (15.1%) patients with myeloma reacted with paratarg-7, a protein of unknown function which is expressed in all human tissues.^24^ Paratarg-7 reactivity was similarly frequent among IgA and IgG paraproteins. In a follow-up study, the authors determined the paraprotein targets in 4 families with familial MGUS or MM.^25,26^ Paraproteins from affected members of two families targeted paratarg-7, and paraproteins from 4 affected members of a fourth family targeted paratarg-8. Paratarg-8 was hyperphosphorylated in the affected family members (pP-8) as is the case with paratarg-7. Six additional autoantigenic non-familial paraprotein targets were also hyperphosphorylated in the patients compared with normal controls, the overall data suggesting that paraproteins of affected members with familial MGUS/MM share family-typical hyperphosphorylated antigens and that hyperphosphorylation of paraprotein targets may represent a common mechanism for development of monoclonal gammopathies. Dhodapkar and colleagues showed that the clonal immunoglobulin in patients with Gaucher’s disease and in mouse models of Gaucher’s disease-associated gammopathy is reactive against lyso-glucosylceramide, which in turn is markedly elevated in these patients and mice. They studied the clonal immunoglobulin in sporadic human monoclonal gammopathies and found that a third of them were specific for lysoglucosylceramide and lysophosphatidylcholine. In fact, reduction in the levels of these lysolipids ameliorated Gaucher’s disease-associated gammopathy in mice, further suggesting a causative association and ascribing a role for long-term immune activation by lysolipids in the development of Gaucher’s disease-associated gammopathies and some sporadic monoclonal gammopathies.

The findings of the current study have several implications. The polyclonal elevation in the immunoglobulin free light chain points toward a general activation of plasma cells or other lymphoid cells that may precede the development of the monoclonal plasma cell population. It strengthens the argument in favor of chronic antigenic stimulation, infectious or otherwise, as a possible initial trigger for the establishment of the MGUS clone. We hypothesize that the first step in the pathogenesis of MGUS is a polyclonal expansion of plasma cells in response to antigenic stimulation that manifests as higher levels of free serum kappa and lambda FLCs. These FLC elevations may be mild enough to not elevate the level of either light chain outside what is considered the normal reference range, but asseen in our study can be detected with greater likelihood of being in the highest population decile of ΣFLC. This polyclonal expansion phase persists for a prolonged period of time, increasing the odds of a plasma cell acquiring one of the two main classes of primary cytogenetic abnormalities (immunoglobulin heavy chain translocations or trisomies) that establish the premalignant MGUS clone. As we have previously described, the MGUS clone once established is at the risk of acquiring additional cytogenetic abnormalities which results in a long-term persistent risk of progression to MM or related malignancy.

From a population standpoint, the identification of biomarkers for development of monoclonal gammopathies can have potential clinical applications in the long term. There is clear evidence suggesting the early intervention in MM at the asymptomatic SMM phase may provide an opportunity to alter the natural history of the disease in a positive way. This is likely to lead to more screening approaches and identification of monoclonal gammopathies in the population, especially in high-risk populations such as first-degree relatives of patients with myeloma, and in blacks. But more importantly, the finding that polyclonal elevations precede the development of MGUS provides clues to etiologic factors. For instance, we have data from our earlier studies that polyclonal elevations of immunoglobulins, as well as MGUS are much more common in Ghana compared with age-matched population in Olmsted County. The findings of this study strengthen the hypothesis that the first step in the pathogenesis of MM in polyclonal expansion of plasma cells presumably in response to antigenic stimulation, and provides important insights for future studies to determine more specific etiologic factors for MGUS and MM.
